# Determining the Best Sensing Coverage for 2-Dimensional Acoustic Target Tracking

**DOI:** 10.3390/s90503405

**Published:** 2009-05-08

**Authors:** Saeid Pashazadeh, Mohsen Sharifi

**Affiliations:** Computer Engineering Department, Iran University of Science and Technology, Tehran, Iran; E-Mail: msharifi@iust.ac.ir

**Keywords:** Wireless sensor networks, quality of service, target tracking, geometric algebra, sensing coverage, accuracy

## Abstract

Distributed acoustic target tracking is an important application area of wireless sensor networks. In this paper we use algebraic geometry to formally model 2-dimensional acoustic target tracking and then prove its best degree of required sensing coverage. We present the necessary conditions for three sensing coverage to accurately compute the spatio-temporal information of a target object. Simulations show that 3-coverage accurately locates a target object only in 53% of cases. Using 4-coverage, we present two different methods that yield correct answers in almost all cases and have time and memory usage complexity of *Θ(1)*. Analytic 4-coverage tracking is our first proposed method that solves a simultaneous equation system using the sensing information of four sensor nodes. Redundant answer fusion is our second proposed method that solves at least two sets of simultaneous equations of target tracking using the sensing information of two different sets of three sensor nodes, and fusing the results using a new customized formal majority voter. We prove that 4-coverage guarantees accurate 2-dimensional acoustic target tracking under ideal conditions.

## 1. Introduction

Surveillance and monitoring of battlefields is one of the most important requirements in critical times. Sometimes it is also quite risky or impossible to have direct surveillance of a real field. That is why for such surveillance tasks remote acoustic target tracking is mostly used. Remote acoustic target tracking can be done by using wireless sensor networks (WSNs). A 2-dimensional acoustic target tracking of a target object by sensed spatio-temporal information obtained using sensor nodes is a 3-dimensional problem. It is often impossible to place wireless sensor nodes in predetermined positions, and this is why in most applications motes (i.e., sensor nodes) are spread randomly in a field from the air. For the sake of simplicity in this paper, we assume that sensor nodes are spread with a uniform distribution in a field and constitute a multi-hop wireless network. Sensor nodes use distributed processing for doing target tracking. In the study reported in this paper we further ignore the signal processing aspects of target tracking using sound sensing.

Quality of service (QoS) plays a critical role on the performability of WSNs that are used for remote target tracking. Attaining the best QoS from the application and end-user's viewpoint though requires the best possible adjustment of WSNs' parameters in support of QoS metrics. This task, namely the QoS management, is the responsibility of WSNs middleware [1]. Providing the best possible values for every QoS metric is difficult because most of the QoS metrics are mutually exclusive. For example, decreases in the sensing coverage increases the network lifetime and at the same time decreases the accuracy and precision of target tracking results [2,3]. One of the challenges in 2-dimensional acoustic target tracking is the determination of the best degree of sensing coverage for accurate target tracking. A high sensing coverage wastes the resources of WSNs while a low sensing coverage decreases the accuracy of target tracking results by producing more frequently happening outliers that greatly decrease the accuracy of the target tracking results.

In this paper we theoretically determine the minimum best sensing coverage of WSNs for 2-dimensional acoustic target tracking that guarantees high accuracy of results without occurrence of outliers. We also present the best possible processing and fusion method that can yield more accurate results with the least sensing coverage and less communication overhead. Since we study the acoustic target tracking from a theoretical point of view in this paper, we ignore the environmental factors like humidity and temperature. The contributions of our paper are applicable to other methods of target tracking like particle filtering [4] and Kalman filtering [5].

The rest of paper is organized as follows. Section 2 presents related work in the area of acoustic target tracking. Section 3 presents the basics of acoustic target tracking and our basic geometric based proposed method, its simulation results and pitfalls. Section 4 discusses the acoustic target tracking from a theoretical perspective and formally represents the different bases of outliers in target tracking and proposes simple solutions for one type of outliers. Section 5 introduces two extended new methods and proves that they eliminate the source of second type of outliers. Section 6 evaluates our proposed methods by reporting the results of our simulations. Section 7 concludes the paper and puts forward some future works.

## 2. Related Work

Basics of sensor nodes positioning for localization and location tracking are discussed in textbooks such as in [6]. Some sensor nodes' localization approaches like ToA [7], TDoA [7] and AoA [8] use the measuring time difference of signal propagation to compute the distances between sensor nodes while some other approaches use the signal strength [6]. If we can measure the distance of a sensor node from a few number of location-aware neighboring nodes, we can evaluate its position using trilateration or multilateration techniques [6]. But in target tracking using sound sensation, the basics of locating a target object is completely different from localization, although there are some similarities too. Using a logical object tracking tree structure for in-network object tracking by considering the topology of network is another approach for target tracking [9]. Organizing sensor nodes as a logical tree facilitates the in-network data processing and reduces the total communication cost of object tracking.

Studies by Wang *et al.* [10] show that acoustic target tracking using WSNs with satisfactory accuracy is possible. They claim three sensing coverage is sufficient for target tracking in 2-dimensional space. The basics of acoustic target tracking are discussed in their paper. Quality ranking is used to decide the quality of tracking results and quality-driven redundancy suppression and contention resolution is effective in improving the information throughput. Like with many other researchers, they assume that all randomly distributed sensor nodes are location-aware [11]. They discuss several factors that influence the accuracy of target tracking and their potential problems. The accuracy of their results is however not excellent and is greatly influenced by the number of location estimation samples. Their studies had shown a higher error margin than it is possible to achieve.

Target tracking studies can be divided into two categories of single target tracking and multiple target tracking. The main aim in multiple target tracking is to separate multiple moving targets from each other [12]. Multiple target tracking using resource limited distributed sensor networks by selecting appropriate fusion mechanism, sensor utility metric and a sensor tasking approach is discussed in [12]. Message-pruning hierarchy trees with the aim of decreasing communication cost and query delay is another approach for multiple target tracking. This method uses a publish-subscribe tracking method [13]. This method is further extended by Lin *et al.* [14] by presenting two message-pruning structures for tracking moving objects and taking into account the physical topology of a sensor network for reflecting real communication cost [14]. They have formulated the object tracking problem as an optimization problem.

Another method for target tracking is the Bayesian framework presented by Ekman *et al.* [4] who have developed particle filters that use data association techniques based on probabilistic data associations. They have studied the tracking of target objects using acoustic sensors that are randomly distributed in a field. Hierarchical classification architecture of sensing subsystem of VigilNet surveillance system is discussed in [15]. This architecture enables efficient information processing for classification and detection of different targets by expanding the processing task in various levels of the system. Using particle filtering is another approach for multiple target tracking [16].

He *et al.* [17] have used WSNs for real-time target tracking with guaranteed deadlines. They have studied the tradeoffs between system properties when meeting real-time constraints. The relations between sensor density and speed of a moving target and wake-up delay of sensor nodes are discussed too. Distributed data association of sensors' measurements are used in multiple target tracking [18]. Hierarchical management and task distribution for target tracking is another proposed management method for WSNs in target tracking applications [19]. Minimizing computational, communication and sensing resources were the main aims in this study.

Using acoustic signal energy measurements of individual sensor nodes to estimate the locations of multiple acoustic sources is another approach for target tracking [20]. They show that a maximum likelihood acoustic source location estimation method compared to existing acoustic energy-based source localization methods yields more accurate results and enhances the capability of multiple source localization.

Using special types of Kalman filtering is another approach to overcome some of the problems of acoustic target tracking such as the problem of global time synchronization [5]. Multiple target tracking are discussed in different scenarios. Multi target tracking has been discussed with two different test case scenarios [21]. In the test cases, sensor nodes are placed in linear and lattice form. Basics of single target tracking and classification and multiple targets that are separated sufficiently in space or time, are discussed in some papers like [22]. They have used a technique called lateral inhibition to reduce computational and network costs while maintaining an accurate tracking.

Beyond studying the various scenarios that are related to locating sensor nodes, some researchers have studied tracking objects with constant velocity with uncertain locations [23]. Using appropriate techniques can cause good time synchronization and localization accuracy that is essential for accurate target tracking [24]. Collaborative signal processing (CSP) is a framework that has been used for tracking multiple targets in a distributed sensor network. The key components include event detection, estimation and prediction of target location, and target classification [25]. Simultaneous localization, tracking and calibration based on Bayesian filter is made in 2-dimensional and 3-dimensional indoor and outdoor spaces with good accurate results [26].

Clearly, there is a rich set of research on the subject of target tracking using WSNs. Several methods like Kalman filtering, statistical methods and numerical methods had been deployed for solving the target tracking problem, but analytical geometric methods had not been used for target tracking. We have opted to use analytical geometric methods in order to attain better tuning of differing QoS parameters that are required and set by end-users at the application-level of WSNs but need to be realized at the middleware-level of WSNs.

## 3. Basics of Acoustic Target Tracking

This section presents the basics of acoustic target tracking in Part 3.1. Part 3.2 proposes a new method based on algebraic geometry for solving simultaneous equations of acoustic target tracking that is originated from the basic model of acoustic target tracking, Part 3.3 presents a simulation model for validating our proposed method, and Part 3.4 shows the results of evaluation of the proposed method.

### 3.1. Acoustic Target Tracking Model

**Figure 1.**
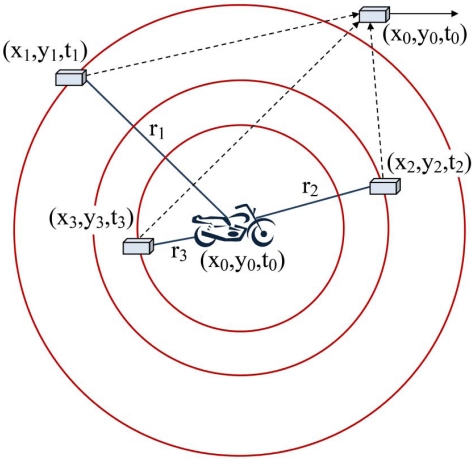
Basic schema of target tracking in 2-dimensional space [27].

We assume that all motes (sensor nodes) have microphones for sensing sound waves. Localization and time synchronization of all motes are done with high accuracy. A target in an unknown location (*x_o_,y_o_*) produces a specific detectable sound wave at time *t_o_*. This sound is broadcasted and reaches the motes after some time delay. The delay depends on the distance of motes from the target object. Figure 1 shows the basic schema of acoustic target tracking in 2-dimensional space by sensing sound waves of a moving target [27]. When a mote *i* senses a sound wave and detects that this sound belongs to a target object of interest, it generates a record consisting of three fields in the form of (*x_i_,y_i_,t_i_*). The first two fields represent the 2-dimensional coordinates of the sensing mote and the third field represents the time at which this mote has sensed the sound of the target object. Each mote broadcasts this record to its neighboring motes in its communication range. The goal is to cooperatively compute the three unknown variables (*x_o_y_o_,t_o_*) of a target object in a distributed way. This record represents the spatio-temporal information of the concerned target object, implying that the target object has generated a sound at time *t_o_* in position (*x_o_y_o_*) that has been detected by motes after some delay. To compute these three unknown variables, we need the sensing information of at least three sensor nodes to create their simultaneous equations.

Based on the Δ*x* = *v*. Δ*t* relation, the distance Δx of a mote from a target object is equal to sound propagation speed (v) times by the time delay from the sensing sound of target object by a mote from the sound generation time of the target object (Δt). The sound propagation speed v is considered 344.0 m/s in this paper. To track a target object, the three unknown variables (*x_o_y_o_,t_o_*) of the target object must be calculated. To calculate these three unknown variables, at least three distinct independent equations are required. In other words, at least three different motes must have detected the sound of a target object at a given position in order to be able to compute the spatio-temporal information of a moving target object. The sensing information of three sensor nodes gives us the simultaneous equations of target tracking in Equation (1):

(1) $$\{\begin{array}{c} \;\sqrt{{\left(x_{o}-x_{1}\right)}^{2}+{\left(y_{o}-y_{1}\right)}^{2}}=\left(t_{1}-t_{o}\right)*v=r_{1}\\ \;\sqrt{{\left(x_{o}-x_{2}\right)}^{2}+{\left(y_{o}-y_{2}\right)}^{2}}=\left(t_{2}-t_{o}\right)*v=\;r_{2}\\ \sqrt{{\left(x_{o}-x_{3}\right)}^{2}+{\left(y_{o}-y_{3}\right)}^{2}}=\left(t_{3}-t_{o}\right)*v=\;r_{3} \end{array}$$

We rewrite simultaneous equations of target tracking and get Equation (2):

(2) $$\{\begin{array}{c} {\left(x-x_{1}\right)}^{2}+{\left(y-y_{1}\right)}^{2}={\left(t_{1}-t\right)}^{2}v^{2}\\ {\left(x-x_{2}\right)}^{2}+{\left(y-y_{2}\right)}^{2}={\left(t_{2}-t\right)}^{2}v^{2}\\ {\left(x-x_{3}\right)}^{2}+{\left(y-y_{3}\right)}^{2}={\left(t_{3}-t\right)}^{2}v^{2} \end{array}$$

Let *C* be a region in 2-dimensional filed. It is said to be *k*-covered if each point in *C* belongs to the sensing range of at least *k* sensors. From this point onward in this paper, coverage is taken synonymous to sensing coverage rather than connectivity.

### 3.2. A New Proposed Method for Solving Simultaneous Equations

The simultaneous equations of target tracking in Equation (2) are degree two and by differencing these equations, as in Equation (3), we can eliminate the degree two factors. This approach is similar to the mathematical solution for the lateration problem in sensor nodes' localization but with major differences [28]. In the lateration problem, the distance of localizing mote from anchor points are known, but in target tracking the distances of target from sensing motes are unknown. The number of unknown variables in target tracking is three but in localization it is two [28]:

(3) $$\{\begin{array}{c} {\left(x-x_{1}\right)}^{2}+{\left(y-y_{1}\right)}^{2}-{\left(x-x_{2}\right)}^{2}-{\left(y-y_{2}\right)}^{2}=({\left(t_{1}-t\right)}^{2}-{\left(t_{2}-t\right)}^{2})v^{2}\\ \begin{array}{c} {\left(x-x_{1}\right)}^{2}+{\left(y-y_{1}\right)}^{2}-{\left(x-x_{3}\right)}^{2}-{\left(y-y_{3}\right)}^{2}=({\left(t_{1}-t\right)}^{2}-{\left(t_{3}-t\right)}^{2})v^{2} \end{array} \end{array}$$

Equation (3) is simplified to:

(4) $$\{\begin{array}{c} 2\left(x_{2}-x_{1}\right)x+2\left(y_{2}-y_{1}\right)y=2\left(t_{2}-t_{1}\right)tv^{2}+\left({t_{1}}^{2}-{t_{2}}^{2}\right)v^{2}+\left({x_{2}}^{2}-{x_{1}}^{2}\right)+\left({y_{2}}^{2}-{y_{1}}^{2}\right)\\ \begin{array}{c} 2\left(x_{3}-x_{1}\right)x+2\left(y_{3}-y_{1}\right)y=2\left(t_{3}-t_{1}\right)tv^{2}+\left({t_{1}}^{2}-{t_{3}}^{2}\right)v^{2}+\left({x_{3}}^{2}-{x_{1}}^{2}\right)+\left({y_{3}}^{2}-{y_{1}}^{2}\right) \end{array} \end{array}$$

Equation (4) is converted into the matrix form as follows:

(5) $$\left[\begin{array}{cc} 2\left(x_{2}-x_{1}\right) & 2\left(y_{2}-y_{1}\right)\\ 2\left(x_{3}-x_{1}\right) & 2\left(y_{3}-y_{1}\right) \end{array}\right]\;\left[\begin{array}{c} x\\ y \end{array}\right]=\left[\begin{array}{c} \begin{array}{c} 2\left(t_{2}-t_{1}\right)v^{2} \end{array}\\ 2\left(t_{3}-t_{1}\right)v^{2} \end{array}\right]\;\left[\begin{array}{c} \begin{array}{c} t \end{array} \end{array}\right]+\left[\begin{array}{c} \left({t_{1}}^{2}-{t_{2}}^{2}\right)v^{2}+\left({x_{2}}^{2}-{x_{1}}^{2}\right)+\left({y_{2}}^{2}-{y_{1}}^{2}\right)\\ \left({t_{1}}^{2}-{t_{3}}^{2}\right)v^{2}+\left({x_{3}}^{2}-{x_{1}}^{2}\right)+\left({y_{3}}^{2}-{y_{1}}^{2}\right) \end{array}\right]$$

Equation (5) is formulated as follows:

(6) $$m\left[\begin{array}{c} x\\ y \end{array}\right]=b\left[\begin{array}{c} t \end{array}\right]+c$$

Equation (6) is solved by using the inverse matrix of *m* as follows [29]:

(7) $$m^{-1}m\left[\begin{array}{c} x\\ y \end{array}\right]=m^{-1}b\left[t\right]+m^{-1}c$$

Equation (7) is further simplified into Equation (8):

(8) $$\left[\begin{array}{c} x\\ y \end{array}\right]=\left[\begin{array}{c} \text{mib}\left(0\right)\\ \text{mib}\left(1\right) \end{array}\right]\;\left[t\right]+\left[\begin{array}{c} \text{mic}\left(0\right)\\ \text{mic}\left(1\right) \end{array}\right]$$

There are only two simultaneous equations in Equation (8), but three unknown variables *x, y* and *t*, so these simultaneous equations have unlimited answers. We need extra equations, so we use the first equation of simultaneous equations of Equation (2) as follows:

(9) $${\left(x-x_{1}\right)}^{2}+{\left(y-y_{1}\right)}^{2}={\left(t_{1}-t\right)}^{2}v^{2}$$

We rewrite Equation (9) in the following form:

(10) $${\left(x-x_{1}\right)}^{2}+{\left(y-y_{1}\right)}^{2}-{\left(t_{1}-t\right)}^{2}v^{2}=0$$

We represent unknown variables *x* and *y* in Equation (8) based on the unknown variable *t* and then substitute them in Equation (10) as follows:

(11) $${\left(\text{mib}(0)t+\text{mic}(0)-x_{1}\right)}^{2}+{\left(\text{mib}(1)t+\text{mic}(1)-y_{1}\right)}^{2}-{\left(t_{1}-t\right)}^{2}v^{2}=0$$

Factorizing Equation (11) with respect to *t*, yields a degree two equation as in Equation (12) that can be solved with delta rule:

(12) $$(\text{mib}{\left(0\right)}^{2}+\text{mib}{\left(1\right)}^{2}-v^{2})t^{2}+\;\left(2\text{mib}\left(0\right)\left(\text{mic}\left(0\right)-x_{1}\right)+2\text{mib}\left(1\right)\left(\text{mic}\left(1\right)-y_{1}\right)+2v^{2}t_{1}\right)t+{\left(\text{mic}\left(0\right)-x_{1}\right)}^{2}+\left(\text{mic}\left(1\right)-y_{1}\right)-{t_{1}}^{2}v^{2}=0$$

Equation (12) can be formulated as follows:

(13) $$at^{2}+bt+c=0$$

The inherent structure of the problem causes the delta of Equation (13) never to become negative. We summarize the result as follows:

(14) $$\begin{array}{c} t=\frac{-b+\sqrt{\Delta }}{2a},\;x=\text{mib}\left(0\right)*t+\text{mic}\left(0\right),\;y=\text{mib}\left(1\right)*t+\text{mic}\left(1\right)\;,\\ t=\frac{-b-\sqrt{\Delta }}{2a},\;x=\text{mib}\left(0\right)*t+\text{mic}\left(0\right),y=\text{mib}\left(1\right)*t+\text{mic}(1) \end{array}$$

Equation (13) gives two different values for variable *t* when delta is greater than zero. Values of *x* and *y* variables are computed by replacing the computed value of *t* variable in Equation (8).

### 3.3. Simulation Model

For our simulations we used the VisualSense simulator [30] that builds on and leverages Ptolemy II [31]. Simulation was done with Ptolemy II version 6.0.2. We used 40 motes with a single sink node. All sensor nodes were spread with normal distribution in 2-dimensional square fields with variation of X position in [0, 500] meters range and Y position in [0, 500] meters range, and the target object rotated 10 times in spiral form in this field passing a unique route in each run such that all parts of simulation field were traversed. Simulation was run for 400 seconds and a target object regularly broadcasted specific acoustic signals in two seconds periods. All sensor nodes were assumed equipped with acoustic sensors, and the acoustic signals of the target object were assumed detectable by all sensor nodes that the target was located in their sensing coverage. Target tracking was carried out 200 times during simulation. The sink had radio communication radius of 240 meters and other motes had an equal radio communication range of 120 meters. We assumed perfect routing without any packet losses.

We used three techniques in our simulations: (1) highly accurate time synchronization with 10^-12^ seconds precision, (2) target tracking by using the information of only three different sensing motes in each set of simultaneous equations, and (3) a simple formal majority voter. We used a variation of formal majority voter presented in [32] for fusing the information of target tracking. The mean of spatially distributed 3-dimensional vectors of spatio-temporal information of the target object was computed first. A nearest vector to the mean vector was chosen as a representative. A vector was then randomly selected from a group of candidate vectors whose Euclidian distance was lower than a specific threshold value (similarity parameter) σ from the representative vector; 0.4 was assigned to the σ parameter.

### 3.4. Summary of Simulation Results

**Figure 2.**
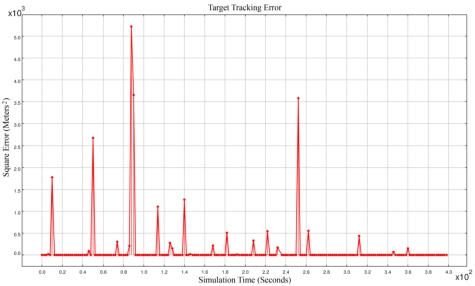
The square errors of target tracking using majority voter.

The accuracy of the best time synchronization algorithms in real cases is in the order of 10^-6^ seconds [33,34]. Our assumed time synchronization accuracy in our simulations (i.e., 10^-12^) is not attainable in real cases; we can consider our simulations to be under ideal perfect time synchronization. Figure 2 shows the square error of target tracking. With this very high time synchronization accuracy, the square error was sometimes in the order of 10^+3^. Simulation results showed that most of the times we had accurate spatio-temporal information of target tracking. But sometimes the network reported results that had big error values. Because these outliers in results were intolerable, we tried to detect the source of this most frequently happening outliers as is described in Section 4.

## 4. Modeling of Acoustic Target Tracking

In Part 4.1 of this section we model and discuss the 2-dimensional acoustic target tracking problem as a geometric problem. In Part 4.2 we then present its dual that is easier to solve and enables us to formally present the sources of outliers in target tracking results. Part 4.3 mathematically describes how acoustic target tracking is done in our method. Part 4.4 introduces the sources of outliers in our method. Part 4.5 mathematically introduces the sources of some outliers in the 2-dimensional acoustic target tracking with three sensing coverage and proposes a simple time test method that can easily eliminate the occurrence of one type of outliers. In Part 4.6 we introduce the second sources of outliers. In Part 4.7 we summarize the simulations results and show the frequency of appearing each type of outliers in target tracking.

### 4.1. Geometric Representation of Acoustic Target Tracking

**Figure 3.**
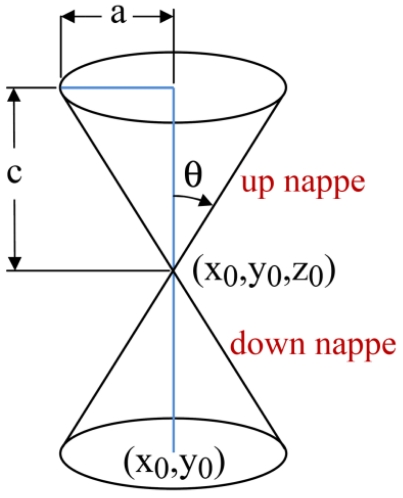
A vertical right circular double cone.

In 2-dimensional space and ideal environmental conditions we can assume that sound propagates in a circular form from a target's location with respect to time. If we consider the third dimension as time, the propagation of sound waves in 2-dimensional space with respect to time makes a vertical circular right cone. Figure 3 shows a vertical right circular double cone, half of its aperture angle, and the coordinates of its apex point. Each piece of double cones that are placed apex to apex is called a nappe.

Equation (15) shows the general equation of a vertical circular right double cone whose apex point has (*x_o_y_o_,z_o_*) coordinate:

(15) $$\frac{{\left(X-x_{o}\right)}^{2}+{\left(Y-y_{o}\right)}^{2}}{a^{2}}=\frac{{\left(Z-z_{o}\right)}^{2}}{c^{2}}$$

*X, Y* and *Z* are free variables in Equation (15), and half of the opening angle of the cone is computed as in Equation (16):

(16) $$\theta =\tan^{-1}(\frac{a}{c})$$

We need to solve simultaneous equations in Equation (2) to compute the time when and the location where a target object had generated a sound. Solving these simultaneous equations can be done using numerical methods. Algebraic geometry can also be used preferably to solve this problem. Algebraic geometry can convert degree two equations to degree one equations and ease the solution of simultaneous equations. It also permits us to visualize and interpret the solution method and results.

We can better represent the target tracking problem as a geometric problem if we rewrite Equation (2) in the form of Equation (17):

(17) $$\{\begin{array}{c} \frac{{\left(x_{1}-x\right)}^{2}+{\left(y_{1}-y\right)}^{2}}{v^{2}}={\left(t_{1}-t\right)}^{2}\\ \frac{{\left(x_{2}-x\right)}^{2}+{\left(y_{2-}y\right)}^{2}}{v^{2}}={\left(t_{2}-t\right)}^{2}\\ \frac{{\left(x_{3}-x\right)}^{2}+{\left(y_{3}-y\right)}^{2}}{v^{2}}={\left(t_{3}-t\right)}^{2} \end{array}$$

**Figure 4.**
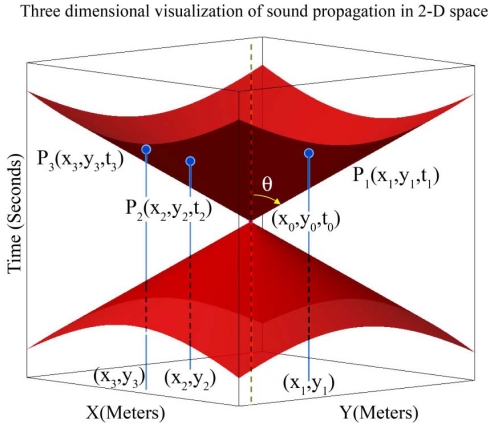
Sound propagation with respect to time in 2-dimensional space.

Let us assume time to be the third dimension that grows in upward direction. Now we can visualize sound propagation in 2-dimensional space with respect to time as a 3-dimensional right circular cone with a specific aperture angle. We have a cone equation and three known points on the surface of its up nappe and we want to determine the coordinate of its apex point that is (*x,y,t*) in Equation (17). Since sound is broadcasted in 2-dimensional space, motes can sense it after some time delay. Motes cannot detect the sound of a target object before the sound is made. For this reason, 3-dimensional sensing information of sensing nodes belongs to the up nappe. Figure 4 shows a double right cone that is generated with sound propagation in 2-dimensional space.

Logically, the up nappe is of interest to target tracking, while degree two equations in simultaneous equations in Equation (2) contain the down nappe too. Aperture of this cone is the angle *2θ* and is related to the sound propagation speed in space and is considered constant and equal to 344.0 m/s in this paper. The aperture of sound propagation cone is calculated as follows:

(18) tan(θ)=v,v=344.0 m/s

Point (*x,y,t*) is the spatio-temporal information of target object and represents the target at time *t* was at position (*x,y*) and has generated a sound. Sensor nodes *i* where *i* = 1,2,3 are distributed on a 2-dimensional surface with known (*x_i_,y_i_*) coordinates. If we draw vertical lines parallel with the time axis, the points at which these lines meet with the surfaces of the up nappe represent the times at which sensor nodes hear the sound of the target object. Sensors can hear the sound of target after some time delay that directly depends on their distances from the target. Information that is sent by each sensor node to its neighboring nodes for computing the spatio-temporal information of the target is a record that can be shown as a point *P_i_* (*x_i_,y_i_,t_i_*) in Figure 4, where *t_i_* represents the time at which sensor *i* hears the sound of the target. Therefore, the geometric representation of the target tracking problem can be stated as: finding the 3-dimensional coordinate of the apex point (*x,y,t*) of a vertical right cone using three known points *P_i_* on the surface of its up nappe.

### 4.2. Dual Geometric Representation of Acoustic Target Tracking

We presented the target tracking problem in the form of a simple geometric problem in Part 4.1. In this part we present the dual of this geometric problem. Solving the dual of this problem is easier than solving the original problem and gives interesting results that will demonstrate the source of outliers in target tracking results.

**Figure 5.**
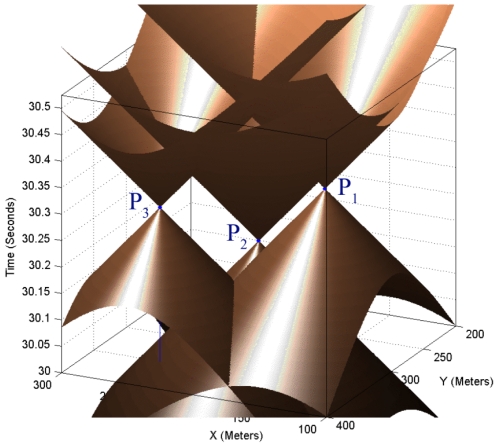
Intersection of three cones for tracking a target object.

The set of equations in simultaneous equations of Equation (2) represent three vertical right circular cones with equal aperture angles whose apex points (*x_i_,y_i_,z_i_*) are different. Reported sensing information of each sensor node is a 3-dimensional point in space that is the 3-dimensional coordinate of the apex point of each cone. In target tracking we look for the points that reside on all of these three cones. Three sensor nodes define three cones that meet each other on some different common points, and only one of them is the real spatio-temporal information of the target object. Figure 5 shows three cones built from the reported information of three sensor nodes.

Figure 5 is the dual of Figure 4 and shows that three sample points on the up nappe of a sound propagation of a target object in Figure 4 can be considered as three vertical unbounded right cones with specific equal aperture angles in Figure 5. Sensor nodes can sense the sound that a target object has generated in the past. Logically, we are only interested in the intersection of three down nappes of motes in Figure 5. Equalities in Equation (2) are degree two equations, so equations of each mote contain up nappe too and up nappes of these sensing cones can thus intersect with each other in a common point. We are not interested in the intersection of up nappes of double cones.

### 4.3. Geometric Solution for Acoustic Target Tracking

#### 

##### Lemma 1

The intersection curve of two vertical right circular cones of sensing information of two different sensor nodes resides on a plane.

##### Proof

Sensing information of each mote *i* in Equation (2) is in the form of a vertical right circular cone as follows:

(19) $${\left(x-x_{i}\right)}^{2}+{\left(y-y_{i}\right)}^{2}={\left(t_{i}-t\right)}^{2}v^{2}$$

Aperture angles of sensing cones of all sensor nodes are equal and can be calculated using Equation (18). By subtracting the equation of cone *j* from equation of cone *i* and by simplification, the following equality is derived:

(20) $$2\left(x_{j}-x_{i}\right)x+2\left(y_{j}-y_{i}\right)y-2\left(t_{j}-t_{i}\right)v^{2}t-\left(\left({t_{i}}^{2}-{t_{j}}^{2}\right)v^{2}+\left({x_{j}}^{2}-{x_{i}}^{2}\right)+\left({y_{j}}^{2}-{y_{i}}^{2}\right)\right)=0$$

All variables *x,y,t* in Equation (20) are in degree one and their coefficients are constant values that are related to location of sensing nodes and the times when they detected the sound of target object. The general form of a plane equation in 3-dimensional space (in *R*^3^) is:

(21) Ax+ By+ Cz+D=0

where vector *N⃗* = 〈*A,B,C*〉 is the normal vector of the plane. Equation (20) is in the form of a plane equation and represents that the intersection curve of two right vertical cones of sensing information of sensor nodes reside on a plane.

##### Definition 1

The intersection of sensing cones of each pair of sensing nodes resides on a plane we call it *the intersection plane*. We denote the intersection plane made by the cones of *i* and *j* sensing nodes by *π_ij_* as in the following equation:

(22) $$\pi_{\text{ij}}:A_{\text{ij}}x+B_{\text{ij}}y+\;C_{\text{ij}}z+\;D_{\text{ij}}=0$$

Cones that are related to sensing nodes are assumed to be unbounded. Three cones of sensing nodes can have three different paired combinations and will have three intersection planes.

##### Definition 2

All planes passing from a common straight line form a *pencil* or *sheaf of planes* and the common straight line is called the *axis of pencil* [35,36].

#### 4.3.1. Geometric Condition of a Pencil Construction

Let us assume that equations of two planes are as follows:

(23) $$\{\begin{array}{c} A_{1}x+B_{1}y+\;C_{1}z+\;D_{1}=0\\ A_{2}x+B_{2}y+\;C_{2}z+\;D_{2}=0 \end{array}$$

Three planes make a pencil if the third plane's equation satisfies the condition of Equation (24) [35]. In other words, if the equation of third intersection plane is a linear combination of equations of two other intersection planes, then these three intersection planes make a pencil:

(24) $$k_{1}(A_{1}x+B_{1}y+\;C_{1}z+\;D_{1})+k_{2}(A_{2}x+B_{2}y+\;C_{2}z+\;D_{2})=0$$

#### 4.3.2. Pencil Condition for Intersection Planes of Target Tracking

##### Lemma 2

The intersection planes of each three sensing cones that are constructed from the sensed information of motes make a pencil.

##### Proof

Assume that our three sensing cones are *i,j,k*. We denote their paired intersection planes as *π_ij_, π_ik_, π_jk_*. The first equation in Equation (25) represents the intersection plane *π_ij_* and the second equation represents the intersection plane *π_ik_*:

(25) $$\begin{array}{c} 2\left(x_{j}-x_{i}\right)x+2\left(y_{j}-y_{i}\right)y-2\left(t_{j}-t_{i}\right)v^{2}t-\left(\left({t_{i}}^{2}-{t_{j}}^{2}\right)v^{2}+\left({x_{j}}^{2}-{x_{i}}^{2}\right)+\left({y_{j}}^{2}-{y_{i}}^{2}\right)\right)=0\\ \begin{array}{c} 2\left(x_{k}-x_{i}\right)x+2\left(y_{k}-y_{i}\right)y-2\left(t_{k}-t_{i}\right)v^{2}t-\left(\left({t_{i}}^{2}-{t_{k}}^{2}\right)v^{2}+\left({x_{k}}^{2}-{x_{i}}^{2}\right)+\left({y_{k}}^{2}-{y_{i}}^{2}\right)\right) \end{array}=0 \end{array}$$

To obtain the equation of the third intersection plane of a pencil, we substitute the equations of planes *π_ij_* and *π_ik_* from Equation (25) in Equation (24) and derive the following:

(26) $$k_{1}\left(2\left(x_{j}-x_{i}\right)x+2\left(y_{j}-y_{i}\right)y-2\left(t_{j}-t_{i}\right)v^{2}t-\left(\left({t_{i}}^{2}-{t_{j}}^{2}\right)v^{2}+\left({x_{j}}^{2}-{x_{i}}^{2}\right)+\left({y_{j}}^{2}-{y_{i}}^{2}\right)\right)\right)+k_{2}\left(\begin{array}{c} 2\left(x_{k}-x_{i}\right)x+2\left(y_{k}-y_{i}\right)y-2\left(t_{k}-t_{i}\right)v^{2}t- \end{array}\left(\left({t_{i}}^{2}-{t_{k}}^{2}\right)v^{2}+\left({x_{k}}^{2}-{x_{i}}^{2}\right)+\left({y_{k}}^{2}-{y_{i}}^{2}\right)\right)\right)=0$$

If we set *k*_1_ = −1 and *k*_2_ = +1 in Equation (26) and simplify it, we get Equation (27), which is the equation of intersection plane *π_jk_* based on Equation (20).

(27) $$2\left(x_{k}-x_{j}\right)x+2\left(y_{k}-y_{j}\right)y-2\left(t_{k}-t_{j}\right)v^{2}t-\left(\left({t_{j}}^{2}-{t_{k}}^{2}\right)v^{2}+\left({x_{k}}^{2}-{x_{j}}^{2}\right)+\left({y_{k}}^{2}-{y_{j}}^{2}\right)\right)=0$$

So the intersection planes of three sensing cones that are constructed from sensed information of motes make a pencil.

##### Definition 3

We call common line of a sheaf of planes as axis of pencil. We denote a pencil that is constructed from intersection planes of three sensing cones *i,j,k* as *ω_ijk_* and axis of this pencil as *ξ_ijk_*.

**Figure 6.**
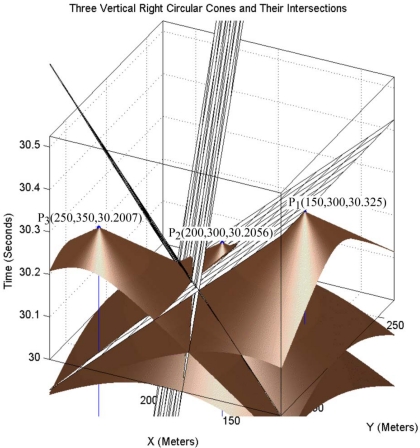
Sensing cones, constructed pencil and axis of pencil.

The equation of two planes of a pencil is sufficient for computing axis of pencil. Furthermore, we saw that the equation of a third intersection plane *π_jk_* is a linear combination of intersection planes *π_ij_* and *π_ik_*. This means that the equation of the third intersection plane *π_jk_* does not provide more information and it is thus redundant. Figure 6 shows the three vertical right circular cones of sensing information of three sensor nodes. This figure also shows the three planes of a pencil, which are the intersection planes of each pair of three cones.

### 4.4. Results of Geometric Solution for Acoustic Target Tracking

#### 

##### Lemma 3

Two sensing cones in a pair intersect with each other on a degree two curve in 3-dimensional space.

##### Proof

We proved in Lemma 1 that the intersection of each pair of sensing cones is a plane. Except special cases, when a plane passes from the apex point of a cone or when a plane lies on the surface of a cone, the intersection of a plane and a cone can produce four different degree two curves [37]. This is proved in many geometric text books like [38] and these curves are known as conic sections.

##### Definition 4

We call a degree two curve that is generated from the intersection of two sensing cones as the *intersection curve* and denote the intersection curve of two sensing cones *i,j* with *σ_ij_*.

##### Theorem 1

Solving simultaneous equations of three different sensing nodes can generate incorrect answer.

##### Proof

As proved in Lemma 1, the intersection curve of two circular right vertical cones in a pair with equal aperture angles reside on a plane. In Lemma 2 we proved that the intersection planes of three sensing cones that are constructed from sensed information of motes make a pencil that intersect on a common line. This is obvious that a line can meet the target tracking cone of Figure 4 in more than one point. One of these two points is the real position of the target. Another point is also mathematically true, but in reality a moving target object cannot be at two different places at once. This is because our basic equations are degree two. So one of the answers is feasible and the other one is incorrect.

**Figure 7.**
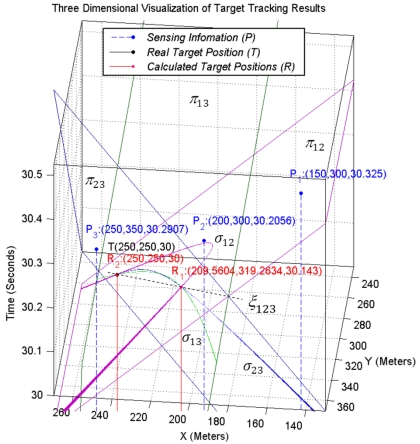
A sheaf of planes and their intersection points with sensing cones at two points.

Figure 7 shows the intersection planes, curves, and axis line of a pencil that is constructed by these intersection planes. As the figure shows, the intersection curve passes from two points that axis plane passes from them too. One of these two points (*R_2_*) is the real spatio-temporal information of the target object and another one (*R_1_*) is its dual and is not the correct result. The real spatio-temporal information of the target object is shown with point *T* in this figure.

### 4.5. Elimination Condition for Second Auxiliary Result

**Figure 8.**
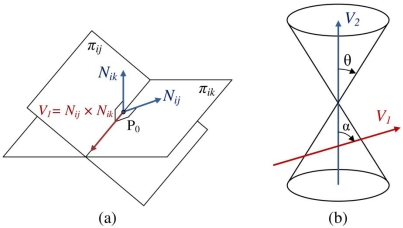
(a) Two planes of a pencil and its axis line. (b) Axis of a pencil and its degree with the axis of cone.

We can eliminate a mathematically correct but unfeasible answer most of the times. If the axis line of pencil crosses the up and the down nappes, then one of the answers belongs to the past and the other one belongs to the future. Sensors cannot hear the sound of a target that is going to be generated in the future. So the points that reside on the intersection point of up nappes in Figure 5 are mathematically correct, but according to our application, they are not feasible answers. We are looking for a condition that the axis of pencil crosses both the up and down nappes in Figure 8b. We will use this condition for eliminating one of the infeasible answers of simultaneous equations in target tracking.

Let *N_ij_* and *N_ij_* be normal vectors of two planes *π_ij_* and *π_ij_* of a pencil as they are shown in Figure 8.a. Vectors *N_ij_* and *N_ik_* are given by equations in Equation (28) that can be extracted from equations of sensing planes in Equation (20):

(28) $$\{\begin{array}{l} N_{\text{ij}}=2\left(x_{j}-x_{i}\right)i+\;2\left(y_{j}-y_{i}\right)j\;-2\left(t_{j}-t_{i}\right)v^{2}k\\ N_{\text{ik}}=2\left(x_{k}-x_{i}\right)i+\;2\left(y_{k}-y_{i}\right)j\;-2\left(t_{k}-t_{i}\right)v^{2}k \end{array}$$

Let us now represent the outer product of these two normal vectors as vector *V*_1_ in Equation (29). This vector is parallel to the axis line of pencil.

(29) $$V_{1}=N_{\text{ij}}\times N_{\text{ik}}$$

Equation (30) represents the vector *V*_1_:

(30) $$V_{1}=(-4(y_{j}-y_{i})(t_{k}-t_{i})v^{2}+4(y_{k}-y_{i})(t_{j}-t_{i})v^{2})i+(-4(t_{j}-t_{i})(x_{k}-x_{i})v^{2}+4(t_{k}-t_{i})(x_{j}-x_{i})v^{2})j+(4(x_{j}-x_{i})(y_{k}-y_{i})-4(x_{k}-x_{i})(y_{j}-y_{i}))k$$

Figure 8.b shows the relative position of an unbounded right vertical cone of sound propagation with vector *V*_1_ that is parallel with the axis line of pencil. Vector *V*_1_ can be represented in a simpler form as:

(31) $$V_{1}=a_{1}.i+b_{1}.j+c_{1}.k\;,\;\{\begin{array}{c} a_{1}=\;\left(\left(y_{k}-y_{i}\right)\left(t_{j}-t_{i}\right)v^{2}-\left(y_{j}-y_{i}\right)\left(t_{k}-t_{i}\right)v^{2}\right)\\ b_{1}=\;\left(\left(t_{k}-t_{i}\right)\left(x_{j}-x_{i}\right)v^{2}-\left(t_{j}-t_{i}\right)\left(x_{k}-x_{i}\right)v^{2}\right)\\ c_{1}=\;\left(\left(x_{k}-x_{i}\right)\left(y_{j}-y_{i}\right)-\left(x_{j}-x_{i}\right)\left(y_{k}-y_{i}\right)\right) \end{array}$$

The axis of a vertical right cone of sound propagation of a target object is a normalized vector as follows:

(32) $$V_{2}=0i+0j+1k$$

The angle between two vectors in 3-dimensional space can be computed using their internal product [39]. In Figure 8.b, the angle between vector *V*_1_ (representing the axis of pencil) and vector *V*_2_ (representing the axis of cone) is shown by *α* and cos(*α*) can be computed as follows:

(33) $$\tau =\cos\left(\alpha \right)=\frac{a_{1}.a_{2}+b_{1}.b_{2}+c_{1}.c_{2}}{\left|V_{1}\right|.\left|V_{2}\right|}=\;\frac{c_{1}}{\sqrt{{a_{1}}^{2}+{b_{1}}^{2}+{c_{1}}^{2}}}$$

The tangent of the angle between the axis of pencil and the axis of sound propagation cone is given by Equation (34):

(34) $$\tan\left(\alpha \right)=\sqrt{\frac{1-\text{cos}{\left(\alpha \right)}^{2}}{\text{cos}{\left(\alpha \right)}^{2}}}=\sqrt{\frac{1-\tau^{2}}{\tau^{2}}}$$

Based on Equation (18), the tangent of half of aperture angle of sound propagation cone is equal to the sound propagation speed that is *V* = 344 *m/s*. Relations in Equation (35) summarize the results of target tracking so far. If the angle between the axis line and the axis of cone is less than the aperture angle of the cone, then this line intersects with the cone in the up and down nappes in two different points and we will have one answer that relates to the past and another one that relates to the future and we can easily eliminate the infeasible answer using a simple test of temporal information of target object. The time at which the target had generated a sound must not exceed the times at which motes had sensed the sound of target object. If the angle between the axis line and the axis of cone is greater than the aperture of the cone, then the axis line intersects with the down nappe in two different points and generates a feasible answer and another infeasible answer that cannot be easily eliminated. If this angle is equal to the aperture of the cone, then this axis line will cut the down nappe in a single point or will fall at the edge of the cone and we will have an unlimited number of position points; in this case, simultaneous equations do not give any answer:

(35) $$\{\begin{array}{l} \begin{array}{c} \text{if}\;\left|\text{tan}\left(\alpha \right)\right|<V\;\text{then}\;\text{one feasible and one infeasible answer}\\ \text{if}\;\left|\text{tan}\left(\alpha \right)\right|=V\;\text{then}\;\text{one feasible and one infinite answers} \end{array}\\ \text{if}\;\left|\text{tan}\left(\alpha \right)\right|>V\;\text{then}\;\text{two feasible answers} \end{array}$$

### 4.6. Source of Abnormal Results

**Figure 9.**
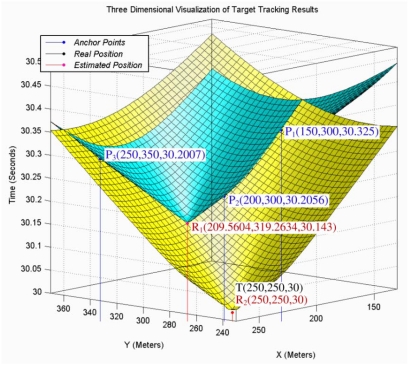
The intersection of axis line of a pencil with down nappe gives two answers where their sound propagation cones pass through three points of sensing information.

When the axis of a pencil intersects with the down nappe in two different points we will have two answers that are mathematically correct, but the target object was located in a specific location and generated sound waves in a specific point of time and only one of these two answers is correct. In this case we cannot use a simple time test to eliminate the incorrect answer. The random selection of one of these two answers by mistake is the source of outliers in target tracking results especially when we have accurate localization and time synchronization. Figure 9 shows this case where the axis line of a pencil intersects only with the down nappe in two different points. The reported information of three individual sensing motes is shown with *P*_1_, *P*_2_ and *P*_3_ points and the target tracking results are shown with *R*_1_ and *R*_2_ points. The real position of target object is shown with point *T*. This figure shows that the sound waves that are broadcasted in the form of cone from the computed coordinates as the spatio-temporal information of target object pass through all sensing points.

### 4.7. Summary of Statistical Results

**Figure 10.**
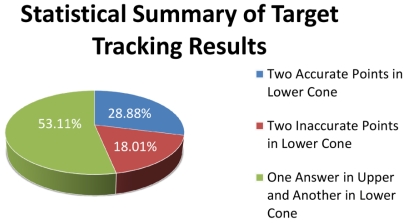
Statistics of different conditions in 2-dimensional target tracking using information of three sensor nodes.

We extensively studied the results of acoustic target tracking with 3-coverage in more than 400 simulations and summarized the results as they are shown in Figure 10. In 53.11% of times we could eliminate the infeasible answer and get the real correct answer. This happened when the axis line of a pencil intersected with the up and down nappes and we could eliminate the infeasible answer using a simple time test.

28.88% of the times we obtained two correct answers, both of which resided on the down nappe, and we could not detect the feasible answer; we randomly chose one of them resulting in the selection of incorrect answers 14.44% of times. In 18.01% of the times that two points resided on the down nappe, one or both answers were inaccurate. This happened when sensing nodes resided very close to a line and error propagation was high. In the remaining parts of this paper we try to eliminate the errors which their source is that the axis line of sensing pencil crossing the down cones in two different points, wherein the correct one is not recognizable.

## 5. Proposed Methods

We proved that solving simultaneous equations of target tracking using information of three sensor nodes always can produce incorrect answers. One type of these outliers that is not under our control is directly related to the specific position of sensor nodes. In this case, we must not consider the results of target tracking because of the high error propagation in such special cases. In this section we present a solution that completely eliminates the generation of outliers in the target tracking results that are related to the weaknesses of our assumptions and methods. We prove that our proposed methods completely solve the problem. In Part 5.1 we propose our first method, called analytic four coverage tracking (AFCT), and prove its correctness, and in Part 5.2 we propose our second method, called redundant answers fusion (RAF), to eliminate outliers in the results.

### 5.1. Modeling AFCT

In this part we introduce the AFCT method, which is the first extension to our basic proposed method that was introduced in Part 3.2. The AFCT method selects the correct spatio-temporal information of a target object when simultaneous equations yield two answers, none of which can be easily eliminated as incorrect with a simple time test. By using four sensing coverage we can solve this problem. Let us assume that a forth mote, in addition to three motes that had sensed the sound of a target, senses the same sound wave of a target object. The sensing information of the fourth mote creates a fourth cone in addition to the previous three cones of Figure 5.

#### 5.1.1. Conditions for Membership of Intersection Planes of Four Sensor Nodes to Make a Pencil

##### Lemma 4

The intersection planes of four sensing cones make a pencil.

##### Proof

In target tracking using the sensing information of three sensor nodes, the equation of two intersection planes is sufficient for calculating the axis of pencil for target tracking. Equation (36) shows the equation of two planes *π*_12_ and *π*_13_.

(36) $$\{\begin{array}{c} 2\left(x_{2}-x_{1}\right)x+2\left(y_{2}-y_{1}\right)y-2\left(t_{2}-t_{1}\right)v^{2}t-\left(\left({t_{1}}^{2}-{t_{2}}^{2}\right)v^{2}+\left({x_{2}}^{2}-{x_{1}}^{2}\right)+\left({y_{2}}^{2}-{y_{1}}^{2}\right)\right)=0\\ 2\left(x_{3}-x_{1}\right)x+2\left(y_{3}-y_{1}\right)y-2\left(t_{3}-t_{1}\right)v^{2}t-\left(\left({t_{1}}^{2}-{t_{3}}^{2}\right)v^{2}+\left({x_{3}}^{2}-{x_{1}}^{2}\right)+\left({y_{3}}^{2}-{y_{1}}^{2}\right)\right)=0 \end{array}$$

Equation (37) shows the intersection planes of the third and fourth cones:

(37) $$\{\begin{array}{c} 2\left(x_{3}-x_{4}\right)x+2\left(y_{3}-y_{4}\right)y-2\left(t_{3}-t_{4}\right)v^{2}t-\left(\left({t_{4}}^{2}-{t_{3}}^{2}\right)v^{2}+\left({x_{3}}^{2}-{x_{4}}^{2}\right)+\left({y_{3}}^{2}-{y_{4}}^{2}\right)\right) \end{array}=0$$

If we can find values for *k*_1_ and *k*_12_ that satisfy Equation (38), we prove that the new intersection plane of Equation (37) makes a pencil with three intersection planes for each pair of three cones of three previous sensing nodes [35].

(38) $$\{\begin{array}{c} x_{3}-x_{4}=k_{1}\cdot (x_{2}-x_{1})+k_{2}\cdot (x_{3}-x_{1})\\ y_{3}-y_{4}=k_{1}\cdot (y_{2}-y_{1})+k_{2}\cdot (y_{3}-y_{1})\\ \left({t_{4}}^{2}-{t_{3}}^{2}\right)v^{2}+\left({x_{3}}^{2}-{x_{4}}^{2}\right)+\left({y_{3}}^{2}-{y_{4}}^{2}\right)=\\ k_{1}\cdot \left(\left({t_{1}}^{2}-{t_{2}}^{2}\right)v^{2}+\left({x_{2}}^{2}-{x_{1}}^{2}\right)+\left({y_{2}}^{2}-{y_{1}}^{2}\right)\right)+k_{2}\left(\left({t_{4}}^{2}-{t_{3}}^{2}\right)v^{2}+\left({x_{3}}^{2}-{x_{4}}^{2}\right)+\left({y_{3}}^{2}-{y_{4}}^{2}\right)\right) \end{array}$$

**Figure 11.**
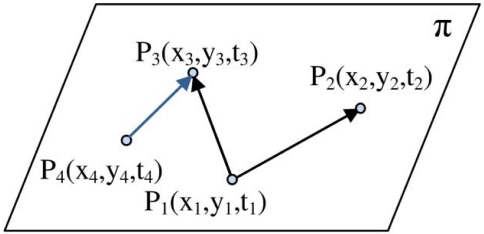
Four points residing on a plane.

The first three equations of simultaneous equations in Equation (38) hold if the normal vector of the new plane is a linear combination of normal vectors of previous planes. Since the sensing cone of the fourth sensing node in Figure 11 satisfies this condition, its intersection planes with other sensing cones belongs to the constructed pencil of previous three sensing cones. If the fourth vector $\vec{P_{3}P_{4}}$ is a linear combination of the three previous vectors, it satisfies the condition of a pencil [40,41]. This occurs when the 3-dimensional sensing information of the fourth sensing node resides on the plane that three previous sensing nodes had made in 3-dimensional space.

The 3-dimensional sensing information of three previous sensor nodes are shown in Figure 11 by *P*_1_, *P*_2_, and *P*_3_ points. Only one plane passes from three points that do not reside on a straight line in the 3-dimensional space. The probability that a random chosen point in the 3-dimensional space resides on a specific plane is nearly zero. Therefore, the probability that the sensing information of a fourth sensor in the form of cone makes intersection planes with three previous cones belonging to the pencil that three cones of three previous sensor nodes had made, is approximately zero.

#### 5.1.2. Properties of the Fourth Sensing Node

**Figure 12.**
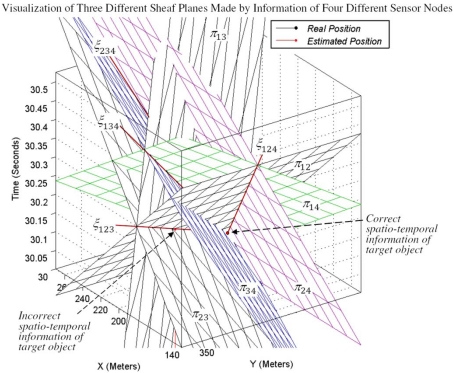
Triple combination of four different sensing motes' information yielding four different pencils that pass through a unique common point.

We proved that the probability that the sensing information of the fourth sensor node belongs to the same pencil of three previous sensor nodes is zero. The information of four different sensing motes that do not reside on the same plane, make four different pencils. Because the coordinates of all four points originated from the same base, triple combination of four sensing motes' information satisfies the condition of a pencil. Four unbounded cones can have six different pairs of combinations with each other that make six intersection planes. Triple combination of sensing information of four sensing nodes makes four different pencils as it is shown in Figure 12.

##### Definition 5

All planes passing through a common point are known as *bundle of planes* [42].

An interesting property of these four pencils is that they all pass through a unique common point most of the times. This point is the correct spatio-temporal information of the target object. As it is shown in Figure 12, uncommon intersection planes of each pencil make a bundle of planes. Figure 12 is an extension of Figure 6 and shows the target tracking results with sensing information of four sensor nodes. Figure 6 shows that only one pencil is generated when we use the information of three individual sensing motes for target tacking. If the axis line of this pencil intersects with the down nappes, there will be two different spatio-temporal information of the target object and the correct one cannot be distinguished. This is also shown in Figure 12 where two answers are located on the *ξ*_123_ axis line, which is the axis line of sheaf of planes *π*_12_, *π*_13_ and *π*_23_ made by the information of three sensing motes 1, 2 and 3. Using the sensing information of a fourth sensing mote helps to distinguish the correct answer. This figure shows that by using the information of a fourth sensing mote, the axis line of four pencils of planes passes through a common point, which is the correct spatio-temporal information of the target object.

**Figure 13.**
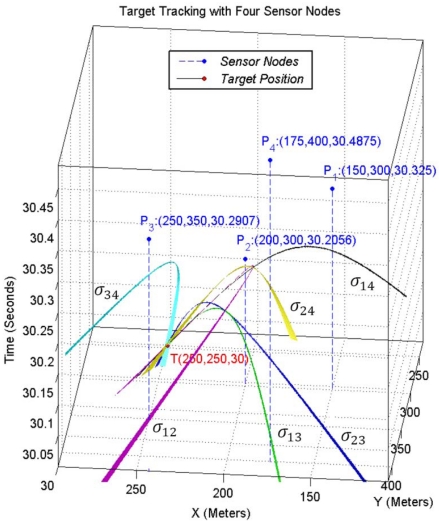
Intersection curves of four sensing cones intersected on a common point.

Figure 13 is the extension of Figure 7. Four sensing cones can have four different triple combinations. All four triple combinations of four cones have three intersection curves that meet each other at two different points. Figure 13 shows that all intersection curves meet each other at one unique common point that represents the real spatio-temporal information of the target object. In 3-coverage target tracking, if the axis line of a pencil intersects with the up and down nappes, we can find the correct answer with 100% confidence. Otherwise, when the axis line of a pencil only intersects with the down nappes, we need the information of a fourth sensing mote, which means we need to have four-sensing coverage. The information of the fourth sensing mote must not reside on the same plane as the planes of the 3-dimensional reported information of three previous sensing motes.

#### 5.1.3. Proving the Properties of Four Sensing Nodes

##### Theorem 2

The use of the sensing information of four sensor nodes in 2-dimensional acoustic target tracking results in a unique correct result.

##### Proof

We proved in Lemma 2 that the intersection planes of three sensing cones make a pencil. Now we extend that work and prove that the intersection planes of four sensing cones make a bundle of planes. We assume that we have four sensing nodes and their pair-wise intersection planes are *π_ij_* where *i,j* ∈ {1,2,3,4}, *i* ≠ *j*. Four sensing cones will have six intersection planes. Four sensing cones can have four triple combinations that make four pencils and so will have four different axis lines. Using Lemma 2, we can conclude that each triple combination of sensing cones of four sensor nodes makes a pencil.

Now we must prove that four different pencils intersect with each other on a common point. Let us assume a pencil *ω_ijk_* that is constructed from the sensing information of sensor nodes *i,j, and k*, and another pencil *ω_ijl_* that is constructed from the sensing information of sensor nodes *i,j, and l*. Pencil *ω_ijl_* consists of three intersection planes *π_ij_, π_il_*, and *π_jl_*, and pencil *ω_ijk_* consists of three intersection planes *π_ij_, π_ik_* and *π_jk_*. Each pair of these pencils has a common intersection plane. The common intersection plane of a pencil *ω_ijk_* and *ω_ijl_* is the *π_ij_* plane. Now we must prove that two uncommon intersection planes of two different pencils make a bundle of planes. We prove that intersection planes *π_il_* and *π_jl_* from the pencil *ω_ijl_* make a bundle of planes with intersection planes *π_ik_* and *π_jk_* of the pencil *ω_ijk_*. Three planes can meet each other on a common point and make a bundle of planes if their equations fall in Equation (39), where (*λ, μ, υ*) ℜ, and their equations are defined up to a common no vanishing factor [42].

(39) $$(A_{1}x+B_{1}y+\;C_{1}z+\;D_{1})+(A_{2}x+B_{2}y+\;C_{2}z+\;D_{2})+\;(A_{3}x+B_{3}y+\;C_{3}z+\;D_{3})=0$$

The equations of intersection planes *π_ij_, π_jl_* and *π_ik_* are as follows, respectively:

(40) $$\{\begin{array}{l} \pi_{\text{il}}:2\left(x_{l}-x_{i}\right)x+2\left(y_{l}-y_{i}\right)y-2\left(t_{l}-t_{i}\right)v^{2}t+\left({t_{i}}^{2}-{t_{l}}^{2}\right)v^{2}+\left({x_{l}}^{2}-{x_{i}}^{2}\right)+\left({y_{l}}^{2}-{y_{i}}^{2}\right)=0\\ \pi_{\text{jl}}:2\left(x_{l}-x_{j}\right)x+2\left(y_{l}-y_{j}\right)y-2\left(t_{l}-t_{j}\right)v^{2}t+\left({t_{j}}^{2}-{t_{l}}^{2}\right)v^{2}+\left({x_{l}}^{2}-{x_{j}}^{2}\right)+\left({y_{l}}^{2}-{y_{j}}^{2}\right)=0\\ \pi_{\text{ik}}:2\left(x_{k}-x_{i}\right)x+2\left(y_{k}-y_{i}\right)y-2\left(t_{k}-t_{i}\right)v^{2}t+\left({t_{i}}^{2}-{t_{k}}^{2}\right)v^{2}+\left({x_{k}}^{2}-{x_{i}}^{2}\right)+\left({y_{k}}^{2}-{y_{i}}^{2}\right)=0 \end{array}$$

We can write the equations of planes in Equation (40) in a simplified form as in Equation (22) and then substitute them in Equation (39) as follows:

(41) $$\lambda .(A_{\text{il}}x+B_{\text{il}}y+C_{\text{il}}z+D_{\text{il}})+.(A_{\text{jl}}x+B_{\text{jl}}y+C_{\text{jl}}z+D_{\text{jl}})+.(A_{\text{ik}}x+B_{\text{ik}}y+C_{\text{ik}}z+D_{\text{ik}})=0$$

If we assume *λ* = −1, *μ* = +1, *and v* = 1 then the above relation is changed to the following:

(42) $$-(A_{\text{ij}}x+B_{\text{ij}}y+C_{\text{ij}}z+D_{\text{ij}})+(A_{\text{ik}}x+B_{\text{ik}}y+C_{\text{ik}}z+D_{\text{ik}})=0$$

Equation (42) is a linear combination of planes *π_ij_* and *π_ik_* equations. We proved in general form that planes *π_ij_, π_ik_*, and *π_jk_* make a pencil. We know that Equation (42) is like Equation (26) and it is the equation of *π_jk_* plane. The condition of Equation (41) thus becomes true and planes of Equation (40) satisfy the condition of Equation (39) and make a bundle of planes.

#### 5.1.4. Applying the AFCT Method

A fusing mote gathers the reported information of all its neighboring motes as well as its own sensed information if it has the sensing capability. It then uses the information of the first three sensing motes to construct the simultaneous equations of Equation (2) and solves these equations to compute the spatio-temporal information of the target object. If the axis line of a pencil intersects in the up and down nappes, then the intersection point of this axis line with the down nappe is the correct spatio-temporal information of the target object. The intersection of axis line of the pencil with up nappes becomes the spatio-temporal information of the target object. But the time of sound generation will be in future in comparison to the time of sensing of target's sound. This is not feasible and we can eliminate it with a simple time test. If the axis line of a pencil intersects only with the down nappes, then the fusing node must use the information of a fourth sensing mote that does not reside on the same plane as the ones constructed from reported information of previous three sensing motes. The fusing node then computes the intersection point of axis lines of two pencils from four pencils. It firstly uses the information of three sensors 1, 2 and 3 to derive two different points as the result. Only one of these two answers falls in the equation of the fourth sensing cone and it is thus the correct answer to acoustic target tracking problem.

#### 5.1.5. Pitfalls of the AFCT Method

**Figure 14.**
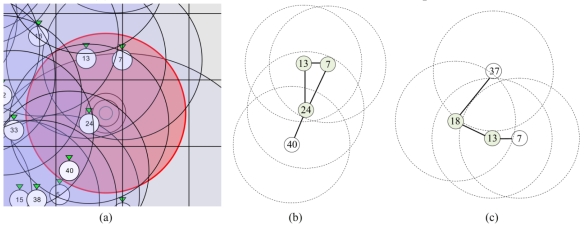
(a) Target tracking in a four sensing coverage area. (b) Connectivity graph of sensor nodes. (c) Connectivity graph of sensor nodes in another part of simulation field.

Figure 14.a shows a part of a simulation field that has 4-coverage for target tracking. Sensor nodes with identifiers 7, 13, 24, and 40 can detect the sound of target object. We assumed that all sensor nodes had the same communication range and all communication links were bidirectional. Figure 14.b shows the connectivity graph of sensor nodes in Figure 14.a. Nodes 7, 13 and 24 can make a set of simultaneous equations with sensing information of three different nodes 7, 13 and 24 to track a target object. In this case a unique set of simultaneous equations can be produced and solved in three different nodes that have high degree of resources; this however consumes resources, especially power, and is somehow against power saving concerns in WSNs that require controlled power awareness and consumption.

Figure 14.c shows the connectivity graph of motes in other parts of simulation area that has four sensing coverage. Nodes 18 and 13 can have the sensing information of three sensor nodes and make a set of simultaneous equations for target tracking. To use four sensing coverage and make a set of simultaneous equations that uses the sensing information of four individual sensing motes, we must have 2-hop sending of sensing information to neighboring nodes and this increases the communication overhead and power consumption. In contrast, node 24 in Figure 14.b can get the sensing information of four different nodes by using a 1-hop communication.

### 5.2. RAF Method

We can use three sensing coverage for accurate target tracking using appropriate local fusion methods. Fusion can be performed locally by the motes that reside on the routing path to the sink node, because local fusion greatly reduces the number of messages to be transmitted and increases the lifetime of the network [43,44]. Local fusion can be performed above the network layer that routing protocols reside [45,46]. Each group of simultaneous equations that is built from the sensing information of three different motes has three types of answers, which were summarized in Equation (35). We can use the confidence level for the information that the WSNs senses and sends to the sink node [47]. If a fusing mote that solves the equation system can determine the correct answer, then it sends the correct target tracking answer with 100% confidence to the sink node. If the fusing node knows that none of the answers are correct, it does not send any result to the sink node. But if it computes two mathematically feasible answers and cannot distinguish which one is the real spatio-temporal information of the target object, it sends both answers to the sink node with 50% confidence for each answer. But if we increase the sensing coverage by 2-hop sending of sensing information, at least two different simultaneous equations can be constructed in two different sensor nodes. For example in Figure 14.c, two different simultaneous equations can be constructed in sensor nodes 18 and 13. In the worst case, each set of simultaneous equations may yield two answers, one of which is correct and the other is incorrect. If we have more redundancy, the number of correct answers relative to incorrect answers increases.

Our proposed method for fusion by intermediate motes in the routing path to the sink node woks as follows. Each mote collects the 3-dimensional spatio-temporal results of reported target tracking information and categorizes the results based on the Euclidean distance similarity in the form of clusters. Then it selects an answer from a group that has more members. This is a special type of formal majority voter that is well suited to fusion by intermediate motes [48-50].

## 6. Simulation Results and Evaluation of Proposed Methods

In this section we compare the simulation results of the AFCT and RAF methods with the basic method proposed in Part 3.2. We also discuss about the cause of peak errors in simulation results of these two extend methods and time and memory usage complexity of them.

### 6.1. Simulation Results of AFCT Method

**Figure 15.**
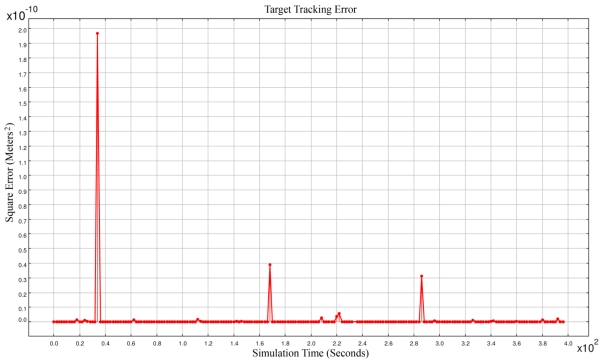
The square error of target tracking using AFCT method.

Figure 15 shows the simulation results when we used the information of four different sensing motes in the required condition mentioned in our AFCT method in Section 5.1. We used a simple formal majority voter like the majority voter in Section 3.3. The maximum error was in the order 10^−10^, implying that in 200 times of target tracking in 400 seconds of simulation run, no target tracking error was encountered. This method uses the sensing information of four sensor nodes in the required conditions. For this purpose, sometimes the sensing information of each sensor node was broadcasted in two hops distance to neighboring sensor nodes.

As Figure 15 shows we have some peak errors in the results. Although the magnitude of this peak errors is in the order of 10^−10^ and ignorable but it shows existence of small outliers. In Section 3.2 we explained our proposed method for solving simultaneous equations. The coefficient matrix of Equation (6) as mentioned earlier is as follows:

(43) $$m=\left[\begin{array}{cc} 2\left(x_{2}-x_{1}\right) & 2\left(y_{2}-y_{1}\right)\\ 2\left(x_{3}-x_{1}\right) & 2\left(y_{3}-y_{1}\right) \end{array}\right]$$

For solving these simultaneous equations we must compute the inverse of this coefficient matrix. If the determinant of this matrix is zero, then this matrix is singular and is not invertible and the system of simultaneous equations of target tracking does not have any answer. This condition holds when the first row of matrix *m* is dependent on the second row, and this condition in turn holds when the three sensing nodes with positions *p*_1_(*x*_1_, *y*_1_), *p*_2_(*x*_2_, *y*_2_) and *p*_3_(*x*_3_, *y*_3_) are located in a straight line. In computation of the inverse matrix *m*^−1^ we need to use the inverse of the determinant of matrix *m*. Determinant of matrix *m* tends to zero when the positions of three sensor nodes tend to reside on a straight line. Therefore the target tracking error increases when the three sensing nodes are close to a straight line. In our simulations all sensor nodes were spread randomly with uniform distribution in a field. When a target passed from a part of the field containing three sensing nodes whose positions were close to a straight line, then the target tracking error increased. We can control this error propagation by adding another condition that if the positions of three sensing node are close to a straight line more than a threshold value, we discard the target tracking results. We did not apply this elimination in our simulations and that is why some peak errors exist in our reported results. We deliberately left out these peaks in our results in order to show that the magnitude of error propagation of our proposed method in ideal conditions is very low. For the same reason we will have peak errors in the RAF method too.

### 6.2. Simulation Results of RAF Method

**Figure 16.**
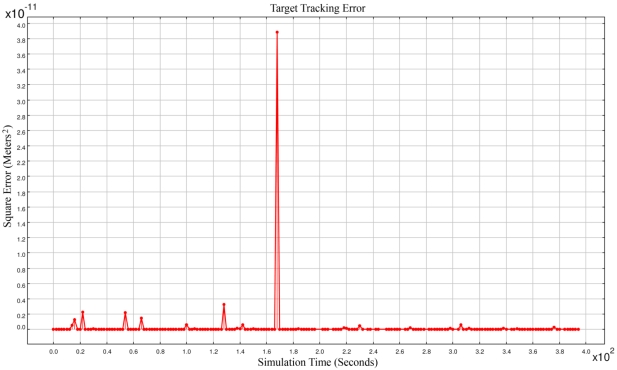
Target tracking error using RAF method.

Figure 16 shows the simulation results when we used our newly proposed fusion method that was presented as the RAF method in Section 5.2. The maximum square error was reduced to 10^−11^ and no incorrect target tracking was encountered in 400 seconds of simulation run. In this method, each set of simultaneous equations was composed of sensing information of three sensor nodes. But for accurate target tracking we needed four sensing coverage. Four sensing coverage caused at least two different sets of simultaneous equations to be generated and using a proper fusion method, accurate spatio-temporal information of a target object to be computed.

AFCT and RAF methods compute the spatio-temporal information of target object without iteration. Time and memory usage complexity of both methods are *Θ(1)*. Other famous methods for acoustic target tracking like particle filtering has time and memory usage complexity of *Θ(n)* which *n* is the number of particles that is used in each step. Typical number of sample particles is about 1000 [4]. Numeric methods like Newton method for solving simultaneous nonlinear equations of Equation (2) are iterative and number of iteration depends on the required accuracy of results and have high computation overhead for application on WSN [51]. In comparison with other methods, our methods are more efficient than other methods form time and memory usage complexity.

## 7. Conclusions and Future Work

### 7.1. Conclusions

Three sensing coverage has been assumed in the literature [52,53] as a sufficient condition for 2-dimensional acoustic target tracking using WSNs. Using geometric representation for modeling, we theoretically proved that three sensing coverage generates outliers in target tracking results and that these outliers occur irrespective of the target tracking method that is used; they occur in target tracking using Kalman filtering, particle filtering and many other methods that assume three sensing coverage is sufficient for 2-dimensional acoustic target tracking.

To reduce the computational overhead of finding the spatio-temporal information of a target object by way of solving quadratic equations, we proposed an alternative method based on geometric algebra that uses linear equations instead of quadratic equations. Our proposed method solves target tracking equations with comparably lower computational overhead and it is applicable to WSNs whose sensor nodes have low processing power. Our simulation results for 2-dimensional target tracking with three sensing coverage in ideal cases, where time synchronization and localization errors were ignored, showed that the target tracking results contain outliers most of the times. Simulation results also showed that in more than half of the cases, three sensing coverage generated outliers in acoustic target tracking. We theoretically proved that four sensing coverage, rather than three sensing coverage, is the necessary condition for accurate 2-dimensional target tracking.

We proposed two other extended methods based on the basic proposed method to improve it, in order to accurately compute the spatio-temporal information of a target object in 2-dimensional space. Both of these methods were based on having four sensing coverage for accurate target tracking. We proved that the AFCT method, which was based on algebraic geometry, accurately computes the spatio-temporal information of a target object with 100% confidence under defined conditions. We also showed through simulation that target tracking errors can be eliminated. The RAF method used a customized fusion method and assumed that each set of simultaneous equations uses the sensing information of only three sensing nodes. But because of four sensing coverage we had at least two different sets of simultaneous equations that allowed us to exploit the capabilities of the second proposed fusion method. Simulation results showed that target tracking was carried out perfectly without any error.

The main contribution of our paper was in formally correcting the belief that *three sensing coverage* is the sufficient condition for accurate acoustic target tracking, by proving that *four sensing coverage* is the necessary condition for accurate acoustic target tracking.

### 7.2. Future work

In real applications of acoustic target tracking, accuracy and precision of target tracking results are two important QoS metrics from view point of the application layer and end user. These metrics are closely related to the time synchronization and localization precision of sensor network. In this paper we discussed about one source of errors that greatly decreases the accuracy of target tracking results. It is difficult to recognize and prove the existence of this source of errors by considering localization and time synchronization errors. Therefore we carried out our studies reported in this paper by assuming very low time synchronization and localization errors.

In real applications, an end user may require different levels of QoS metrics based on the state of the system. If the end user wants to have control of the accuracy and precision of target tracking results, it is necessary to know the factors that influence the accuracy and precision of target tracking results and also know the relationship between influencing factors and the accuracy and precision of target tracking results. Knowing these, it will be possible for a middleware to guarantee the required level of accuracy and precision of target tracking errors by adjusting the time synchronization and localization precision parameters. To achieve this, the error propagation of our proposed method is currently under further study.

## References

[1] Molla M.M., Ahmad S.I. A Survey of Middleware for Sensor Network and Challenges.

[2] Wan P.J., Yi C.W. (2006). Coverage by Randomly Deployed Wireless Sensor Networks. IEEE Trans. Inf. Theory.

[3] Intanagonwiwat C., Estrin D., Govindan R., Heidemann J. Impact of Network Density on Data Aggregation in Wireless Sensor Networks.

[4] Ekman M., Davstad K., Sjoberg L. Ground target tracking using acoustic sensors.

[5] Tsiatsis V., Srivastava M.B. The Interaction of Network Characteristics and Collaborative Target Tracking in Sensor Networks.

[6] Tseng Y.C., Huang C.F., Kuo S.P., Ilyas M., Mahgoub I. (2005). Positioning and Location Tracking in Wireless Sensor Networks. Handbook of Sensor Networks: Compact Wireless and Wired Sensing Systems.

[7] Savvides A., Han C.C. Srivastava, M. Dynamic Fine-Grained Localization in Ad-Hoc Networks of Sensors.

[8] Niculescu D., Nath B. (2003). Ad hoc Positioning System (APS) Using AoA.

[9] Lin C.Y., Peng W.C., Tseng Y.C. (2006). Efficient In-Network Moving Object Tracking in Wireless Sensor Networks. IEEE Trans. Mobile Computing.

[10] Wang Q., Chen W.P., Zheng R., Lee K., Sha L. Acoustic Target Tracking Using Tiny Wireless Sensor Devices.

[11] Gupta R., Das S.R. Tracking moving targets in a smart sensor network.

[12] Liu J., Chu M., Reich J.E. (2007). Multitarget tracking in distributed sensor networks. IEEE Signal Proc. Mag..

[13] Kung H.T., Vlah D. Efficient location tracking using sensor networks.

[14] Lin C.Y., Tseng Y.C. Structures for In-Network Moving Object Tracking in Wireless Sensor Networks.

[15] Gu L., Jia D., Vicaire P., Yan T., Luo L., Tirumala A., Cao Q., He T., Stankovic J., Abdelzaher T., Krogh B. Lightweight Detection and Classification for Wireless Sensor Networks in Realistic Environments.

[16] Hue C., Le Cadre J.-P., Perez P. (2002). Tracking multiple objects with particle filtering. IEEE Trans. Aerosp. Electron. Syst..

[17] He T., Vicaire P.A., Yan T., Luo L., Gu L., Zhou G., Stoleru R., Cao Q., Stankovic J.A., Abdelzaher T. Achieving Real-Time Target Tracking Using Wireless Sensor Networks.

[18] Chen L., Cetin M., Willsky A.S. Distributed data association for multi-target tracking in sensor networks.

[19] Guibas L.J. (2002). Sensing, Tracking, and Reasoning with Relations. IEEE Signal Proc. Mag..

[20] Sheng X., Hu Y.H. (2005). Maximum Likelihood Wireless Sensor Network Source Localization Using Acoustic Signal Energy Measurements. IEEE Trans. Signal Process..

[21] Holmqvist T. (2006). Person Tracking using Distributed Sensor Networks. M.Sc Thesis.

[22] Brooks R.R., Ramanathan P., Sayeed A.M. (2003). Distributed Target Classification and Tracking in Sensor Networks. Proc. IEEE.

[23] Barsanti R.J., Tummala M. Combined Acoustic Target Tracking and Sensor Localization.

[24] Girod L., Bychkovskiy V., Elson J., Estrin D. Locating tiny sensors in time and space: A case study.

[25] Dan L., Wong K. D., Yu H. H., Sayeed A.M. (2002). Detection, classification, and tracking of targets. IEEE Signal Process. Mag..

[26] Taylor C., Rahimi A., Bachrach J., Shrobe H. Simultaneous Localization, Calibration, and Tracking in an ad Hoc Sensor Network.

[27] Pashazadeh S., Sharifi M. Simulative Study of Target Tracking Accuracy Based on Time Synchronization Error in Wireless Sensor Networks.

[28] Karl H., Willig A. (2005). Protocols and Architectures for Wireless Sensor Netweorks.

[29] Nicholson W.K. (1995). Linear Algebra with Applications.

[30] Baldwin P., Kohli S., Lee E.A., Liu X., Zhao Y. Visualsense: Visual Modeling for Wireless and Sensor Network Systems.

[31] Baldwin P., kohli S., Lee E.A., Liu X., Zhao Y. Modeling of Sensor Nets in Ptolemy II.

[32] Lorczak P.R., Caglayan A.K., Eckhardt D.E. (1989). A Theoretical Investigation of Generalized Voters for Redundant Systems.

[33] Ganeriwal S., Kumar R., Adlakha S., Srivastava M. (2003). Network-Wide Time Synchronization in Sensor Networks. NESL Technical Report, NESL 01-01-2003.

[34] Ganeriwal S., Kumar R., Srivastava M.B. Timing-Sync Protocol for Sensor Networks.

[35] Woods F.S. (1922). Higher Geometry: An Introduction to Advanced Methods in Analytic Geometry.

[36] Gellert W., Gottwald S., Hellwich M., Kästner H., Künstner H. (1989). VNR Concise Encyclopedia of Mathematics.

[37] Gutenmacher V., Vasilyev N.B. (2004). Lines and Curves: A Practical Geometry Handbook.

[38] Gibson C.G. (2003). Elementary Euclidean geometry: an Introduction.

[38] Vince J. (2006). Mathematics for Computer Graphics.

[39] Vince J. (2008). Geometric Algebra for Computer Graphics.

[40] Gruenberg K.W., Weir A.J. (1977). Linear Geometry.

[41] Vaisman I. (1997). Analytical Geometry.

[42] Liang B., Liu Q. A Data Fusion Approach for Power Saving in Wireless Sensor Networks.

[43] Yamamoto H., Ohtsuki T. (2005). Wireless sensor networks with local fusion.

[44] Farivar R., Fazeli M., Miremadi S.G. Directed flooding: a fault-tolerant routing protocol for wireless sensor networks.

[45] Intanagonwiwat C., Govindan R., Estrin D. Directed Diffusion: A Scalable and Robust Communication Paradigm for Sensor Networks.

[46] Sharifi M., Pashazadeh S. Using Confidence Degree for Sensor Reading in Wireless Sensor Networks.

[47] Pullum L.L. (2001). Software Fault Tolerance - Techniques and Implementation. Artech House.

[48] Iyengar S.S., Prasad L. (1995). A general computational framework for distributed sensing and fault-tolerant sensor integration. IEEE Trans. syst. Man Cybern..

[49] Broen R.B. (1975). New Voters for Redundant Systems. J. Dyn. Syst. Meas. Control.

[50] Süli E., Mayers D.F. (2003). An introduction to numerical analysis.

[51] Cheng C.-T., Tse C.K., Lau F.C.M. A Bio-Inspired Scheduling Scheme for Wireless Sensor Networks.

[51] Kim J.E., Yoon M.K., Han J., Lee C.G. Sensor Placement for 3-Coverage with Minimum Separation Requirements.

